# Expanding the Functional Scope of the Fmoc‐Diphenylalanine Hydrogelator by Introducing a Rigidifying and Chemically Active Urea Backbone Modification

**DOI:** 10.1002/advs.201900218

**Published:** 2019-04-19

**Authors:** Vasantha Basavalingappa, Tom Guterman, Yiming Tang, Sivan Nir, Jiangtao Lei, Priyadarshi Chakraborty, Lee Schnaider, Meital Reches, Guanghong Wei, Ehud Gazit

**Affiliations:** ^1^ Department of Molecular Microbiology and Biotechnology George S. Wise Faculty of Life Sciences Tel Aviv University Tel Aviv 69978 Israel; ^2^ Department of Physics State Key Laboratory of Surface Physics Key Laboratory for Computational Physical Sciences (MOE), and Collaborative Innovation Center of Advanced Microstructures (Nanjing) Fudan University Shanghai 200433 P. R. China; ^3^ Institute of Chemistry The Hebrew University of Jerusalem Jerusalem 91905 Israel

**Keywords:** anion sensing, antifouling materials, metal nanoparticles, peptide self‐assembly, peptidomimetics, urea slow release, ureidopeptides

## Abstract

Peptidomimetic low‐molecular‐weight hydrogelators, a class of peptide‐like molecules with various backbone amide modifications, typically give rise to hydrogels of diverse properties and increased stability compared to peptide hydrogelators. Here, a new peptidomimetic low‐molecular‐weight hydrogelator is designed based on the well‐studied *N*‐fluorenylmethoxycarbonyl diphenylalanine (Fmoc‐FF) peptide by replacing the amide bond with a frequently employed amide bond surrogate, the urea moiety, aiming to increase hydrogen bonding capabilities. This designed ureidopeptide, termed Fmoc—Phe—NHCONH—Phe—OH (Fmoc‐FuF), forms hydrogels with improved mechanical properties, as compared to those formed by the unmodified Fmoc‐FF. A combination of experimental and computational structural methods shows that hydrogen bonding and aromatic interactions facilitate Fmoc‐FuF gel formation. The Fmoc‐FuF hydrogel possesses properties favorable for biomedical applications, including shear thinning, self‐healing, and in vitro cellular biocompatibility. Additionally, the Fmoc‐FuF, but not Fmoc‐FF, hydrogel presents a range of functionalities useful for other applications, including antifouling, slow release of urea encapsulated in the gel at a high concentration, selective mechanical response to fluoride anions, and reduction of metal ions into catalytic nanoparticles. This study demonstrates how a simple backbone modification can enhance the mechanical properties and functional scope of a peptide hydrogel.

Peptide hydrogels are solid‐like, biocompatible, and biodegradable supramolecular materials, which are hence especially suitable for biological and biomedical applications such as 3D cell culture, tissue engineering, and controlled drug release.^1^, ^2^ Peptide hydrogels can also be utilized for other applications, such as sensing, catalysis, and optoelectronics, in which they act as structural scaffolds or directly perform desired functions.^3^ Yet, the application of peptide hydrogels is still limited in many cases due to poor or marginally tunable rheological properties, lack of enzymatic stability, and absence of multifunctionality.^4^, ^5^, ^6^ Moreover, in the process of generating application‐specific functionalities by mutating the amino acid sequence, the mechanical properties of peptide hydrogels may become impaired.^7^, ^8^, ^9^ A recently emerging strategy to address these issues is the design of peptidomimetic hydrogelators, in which one or more of the typical amide bonds have been replaced by other chemical groups that tether the constituent amino acids. This molecular design has led to metabolically stable nanostructures with tuneable mechanical properties, which serve as platforms in therapeutic and biomedical applications.^4^, ^10^, ^11^ Such studied peptidomimetic hydrogelators include depsipeptides,^12^ oxazolidine‐tethered,^13^ and cyclobutane‐tethered peptides,^14^ which form hydrogels of improved rigidity and longer biostability compared to their native α‐peptide counterparts. Here, we aimed to further explore the beneficial effect of backbone modification on hydrogel properties and functionalities. To this end, we selected the well‐studied *N*‐fluorenylmethoxycarbonyl diphenylalanine (Fmoc‐FF) hydrogelator as a model system.^15^, ^16^, ^17^, ^18^, ^19^ Several approaches, such as co‐assembly, covalent modification of the side chain or protecting group, variation of solvent composition, and pH modulations, have been employed in multiple studies to fine‐tune the physical properties of the Fmoc‐FF hydrogel.^17^, ^19^, ^20^, ^21^, ^22^, ^23^, ^24^, ^25^ Yet, to our knowledge, its backbone modification has been attempted only twice by modifying the Fmoc‐FF amide group into *N*‐benzyl glycine (Nphe) or ester. These modifications resulted in decreased hydrogen bonding, which led, in turn, to decreased rigidity of the formed hydrogel, as compared to the Fmoc‐FF under the same conditions.^26^, ^27^ Accordingly, it would be expected that increasing the hydrogen bonding capability of the hydrogelator by the incorporation of a urea moiety would enhance the rigidity of its hydrogels. Indeed, a related congruent report has shown that urea‐based nonpeptidic gelators self‐associate through N—H/O hydrogen bonds to form stable six‐membered rings based on two donors and one carbonyl acceptor, thereby resulting in stronger urea–urea α‐tape hydrogen bonding interactions.^28^, ^29^ Moreover, the presence of a urea moiety could impart self‐assembled nanostructures with novel functional properties or increased mechanical strength and metabolic stability, as compared to their unmodified counterparts.^29^, ^30^, ^31^ For these reasons, we selected the urea group as a backbone modification for Fmoc‐FF.

We report the incorporation of a urea moiety such that it substitutes the amide bond of Fmoc‐FF to form Fmoc—Phe—NHCONH—Phe—OH (Fmoc‐FuF, **Figure** **1**a). Fmoc‐FuF proved to be an efficient hydrogelator, giving rise to a hydrogel of improved rigidity, as compared to the unmodified Fmoc‐FF hydrogel. The Fmoc‐FuF hydrogel also exhibited self‐healing, shear thinning, and in vitro biocompatibility. In terms of functionality, substrates coated with the Fmoc‐FuF xerogel reduced the accumulation of bacteria, demonstrating rudimentary antifouling properties. Furthermore, free urea was encapsulated at a high concentration within the Fmoc‐FuF hydrogel network, resulting in slow release of the urea into the environment and hence suggesting a potential application of Fmoc‐FuF in slow‐release urea fertilizers. Fmoc‐FuF also showed characteristics of nonpeptidic urea‐based gelators, such as mechanical responsiveness to anion stimuli and reduction of metal ions into nanoparticles. Importantly, these functional properties were not displayed by the unmodified Fmoc‐FF. Overall, we developed a multifunctional aromatic ureidopeptide hydrogelator of low molecular weight with potential envisioned applications in tissue engineering, chemical catalysis, antifouling, and agriculture.

**Figure 1:**
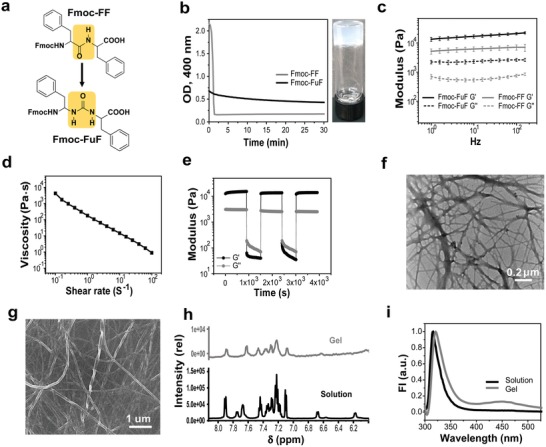
Characterization of the Fmoc‐FuF hydrogel. a) Chemical structure of Fmoc‐FF and Fmoc‐FuF hydrogelators, highlighting (yellow) the unmodified (Fmoc‐FF) or modified (Fmoc‐FuF) backbone. b) Left: OD kinetics at 400 nm. Right: Photograph of the semitransparent Fmoc‐FuF hydrogel. c) Frequency sweep measurement for Fmoc‐FuF and Fmoc‐FF hydrogels. Data represent mean ± standard deviation (SD, *n* = 3 hydrogels per condition). d) Flow sweep measurement showing shear‐thinning behavior of Fmoc‐FuF hydrogel. e) Five‐step loop time sweep measurement showing the thixotropic nature of Fmoc‐FuF hydrogel. f) TEM and g) HRSEM images of Fmoc‐FuF fibers. h) Partial ^1^H NMR spectrum, showing the NH and aromatic regions, in the solution and gel states. i) Fluorescence emission spectrum of Fmoc‐FuF in solution and gel states (λ_ex_ = 285 nm).

The Fmoc‐FuF hydrogel was prepared using the solvent‐switch method by diluting a dimethyl sulfoxide (DMSO) stock solution of Fmoc‐FuF with water to a final concentration of 0.5 wt% and 20% (v/v) DMSO. Thus, a turbid Fmoc‐FuF solution had formed, which transitioned within 1 min into a semitransparent hydrogel that became more optically clear over a period of 30 min as per turbidometry (Figure 1b; Figure S1, Supporting Information). Compared with Fmoc‐FuF, a control of Fmoc‐FF presented higher initial solution turbidity, slightly slower gelation time (≈90 s), and considerably faster rate of optical clearance (Figure 1b; Figure S1, Supporting Information), suggesting distinct gelation dynamics for the two hydrogelators.

The Fmoc‐FuF hydrogel is elastic, as was evident by rheological measurements. Oscillatory strain sweep (0.1–300%; Figure S2, Supporting Information) and frequency sweep (0.1–100 Hz; Figure 1c) showed that in the linear viscoelastic region, the storage modulus (*G*′) of the Fmoc‐FuF hydrogel is an order of magnitude higher than its loss modulus (*G*″), characteristic of elastic hydrogels.^32^ Interestingly, the *G*′ value (at 10 Hz) of the Fmoc‐FuF hydrogel is 2.7‐fold higher than that of Fmoc‐FF at the same wt% value (Figure 1c). In line with this observation, oscillatory strain measurements at a constant frequency of 1 Hz showed that the critical strain value γ, required for gel breaking, is higher by 20% for Fmoc‐FuF, as compared to Fmoc‐FF at the same wt% value (Figure S2a,b, Supporting Information). These data therefore show that the urea backbone modification results in the improvement of the hydrogel mechanical properties, as was also reported for a nonpeptidic hydrogelator.^31^ In this context, it is also worth mentioning the reported effect of pH on the mechanical properties of Fmoc‐FF gels,^19^ where higher pH, especially above 5, leads to lower *G*′. Interestingly, the measured pH of Fmoc‐FuF hydrogels (5.2 ± 0.1) is higher than that of control Fmoc‐FF gels (4.7 ± 0.5) and yet *G*′ of the former hydrogel is higher. Hence, the urea backbone modification, while leading to a higher gel pH, overall improves the mechanical properties of the hydrogel. We note that although the incorporation of a backbone urea group is expected to improve the hydrogel mechanical properties by enhancing hydrogen bonding, its beneficial effect may be exerted by additional mechanisms, such as modulation of other noncovalent interactions.

Rheologically, Fmoc‐FuF hydrogel presented shear‐thinning behavior (Figure 1d), similar to that of Fmoc‐FF, in line with a previous report on Fmoc‐FF^33^ (Figure S2c, Supporting Information). Fmoc‐FuF hydrogels additionally showed thixotropic and self‐healing properties.^33^ A five‐step loop time sweep test, at a low strain of 0.1% (*G*′ > *G*″, gel state) and a high strain of 500% (*G*″ > *G*′, solution state),^32^ demonstrated the thixotropic nature of Fmoc‐FuF although a 10% decrease in *G*′ was observed as compared with the first step (Figure S2d, Supporting Information). Similar thixotropic behavior, with a smaller decrease in *G*′ (<2%), was observed for Fmoc‐FF hydrogels (Figure S2e,f, Supporting Information). The self‐healing property of the Fmoc‐FuF hydrogel was demonstrated by cutting and rejoining two hydrogel monoliths, which then bridged a 2.5 cm long elevated gap, were lifted vertically from the surface using forceps, and after 2 h did not show visible cut marks (Figure S3a–c, Supporting Information). Similarly, Fmoc‐FF rejoined hydrogel bridged an elevated gap, yet it could not be lifted using forceps (Figure S3d–f, Supporting Information). Similar to the rejoined Fmoc‐FuF hydrogel, rejoined Fmoc‐FF hydrogel did not show visible cut marks after 2 h.

We next investigated the underlying nanoscale morphology and molecular organization of Fmoc‐FuF in the gel state. Morphologically, transmission electron microscopy (TEM) and high‐resolution scanning electron microscopy (HRSEM) revealed that the Fmoc‐FuF hydrogel consists of typically entangled, flat, and twisted fibers 10–20 nm in width (Figure 1f,g). Complementing atomic force microscopy (AFM) imaging showed that the fiber height ranges from 10 to 30 nm (Figure S4, Supporting Information). At the molecular level, ^1^H NMR spectroscopy showed significant line broadening of the signals for the gel state as compared with the sharp peaks of dissolved Fmoc‐FuF (Figure 1h). This change is associated with a reduction in molecular‐scale mobility of the gelator and its conversion to a solid‐like state upon molecular self‐assembly.^34^, ^35^ Since peaks associated with NH protons almost disappeared in the gel versus the dissolved state, a hydrogen bonding network presumably forms upon the self‐assembly of Fmoc‐FuF into fibers in the gel state. Further structural insights were gained by fluorescence spectroscopy. In the emission spectrum (300–500 nm, λ_ex_ = 285 nm), the characteristic fluorenyl ring band showed a redshift from 315 to 321 nm in the gel versus dissolved state, likely due to the formation of fluorenyl excimer with antiparallel arrangement and π–π stacking of the fluorenyl group.^36^ The tail in the visible region at 455 nm is indicative of the presence of an extensive *J*‐aggregate, which may include both phenyl and fluorenyl rings^36^ (Figure 1i).

To elucidate the structural features and self‐assembly mechanism of Fmoc‐FuF assemblies at the molecular level, we performed microsecond‐long coarse‐grained molecular dynamics (CG‐MD) simulations on systems consisting of 200, 400, or 600 Fmoc‐FuF molecules in aqueous solutions containing 20% (v/v) DMSO (**Figure** **2**a). In the simulation of the two larger systems, starting from disordered states, the molecules first aggregated into small spherical clusters and large worm‐like irregular aggregates (*t* = 0.1 µs), which then started to fuse into a large branched aggregate showing the structural characteristics of gels.^37^ This process was not observed in the smaller system.

**Figure 2:**
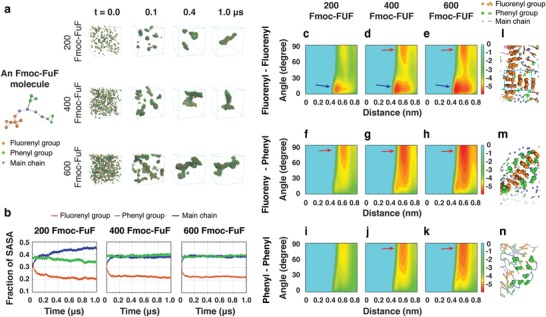
Self‐assembly mechanism of Fmoc‐FuF revealed by microsecond‐long CG‐MD simulations of 200, 400, and 600 molecules. a) Snapshots of aggregates at four timepoints. The CG representation of an Fmoc‐FuF molecule is shown for clarity. b) Time evolution of the SASA fraction of fluorenyl, phenyl, and main chain groups. c–k) The FEL of worm‐like and branched gel‐like assemblies as a function of the centroid distance and the angle between the two aromatic rings in three different ring pairs. The basins are marked by arrows. l–n) Stacking patterns of fluorenyl–fluorenyl, fluorenyl–phenyl, and phenyl–phenyl ring pairs.

The roles of the fluorenyl, two phenyls, and main chain groups in the self‐assembly process were assessed by the fraction of their solvent accessible surface area (SASA; Figure 2b; Figure S5, Supporting Information). The SASA fraction of the fluorenyl group rapidly dropped within the first 0.1 µs and started fluctuating at around 0.2 µs, whereas the SASA fraction of the main chain quickly increased, reaching a plateau. Interestingly, the SASA fraction of the phenyl group did not significantly change throughout the simulation. These data indicate that the fluorenyl group is mostly buried inside the aggregate forming the hydrophobic spine, while the main chain is generally solvent‐exposed. Additionally, the fluorenyl group appears to play a crucial role in the formation of the branched Fmoc‐FuF aggregates. To further examine the importance of aromatic stacking in the self‐assembly process,^36^, ^38^, ^39^ free energy landscape (FEL) as a function of the centroid distance and the angle of the fluorenyl–fluorenyl, fluorenyl–phenyl, and phenyl–phenyl aromatic ring pairs was calculated (Figure 2c–k). The basin located at 10° (0.45 nm) of angle (centroid distance) for the 200/400/600 systems indicated a strong preference for parallel stacking between fluorenyl rings in the nonbranched worm‐like aggregate (Figure 2c–e). The additional basin at 80°, 0.45 nm, in the 400/600 systems (Figure 2d,e) indicated that besides parallel, perpendicular (T‐shaped) stacking patterns are also preferred. The difference between Figure 2c–e reveals the importance of T‐shaped stacking in the formation of branched gel‐like aggregates (Figure 2l). A shallow minimum‐energy basin at 80°, 0.6 nm, in the FEL of the 200‐Fmoc‐FuF system (Figure 2f,i) corresponded to parallel stacking of fluorenyl–phenyl and phenyl–phenyl rings. This was much deeper and larger in the 400/600 systems (Figure 2g,h,j,k), corresponding to T‐shaped, herringbone, and parallel stacking patterns (Figure 2m,n). These results revealed different stacking pattern preferences in nonbranched worm‐like and branched gel‐like aggregates, and the crucial role of the T‐shaped fluorenyl–fluorenyl stacking pattern in the formation of branched gel‐like aggregates. Interestingly, the π–π stacking interaction of Fmoc groups also plays an important role in stabilizing the Fmoc‐AA supramolecular assemblies, as reported in a recent MD simulation study by Mu et al.^40^

Following structural investigation, we explored five functionalities of Fmoc‐FuF and its hydrogel, in which we compared the performance of Fmoc‐FuF to that of Fmoc‐FF. First, the anion binding of Fmoc‐FuF was explored. Urea‐based compounds are prominent anion receptors, which bind anions in a specific manner via two directional hydrogen bonds.^29^, ^41^, ^42^ In the case of Fmoc‐FuF, specific interaction with fluoride ions (F^−^) generated blue fluorescence, attributed to the formation of a charge‐transfer complex^42^ (**Figure** **3**a). Blue fluorescence upon UV illumination was observable to the naked eye when an Fmoc‐FuF DMSO solution was supplemented with F^−^, but not with other anions (Figure 3b, a 100 × 10^−3^ m pH of ≈7 stock solution of tetrabutylammonium fluoride, TBAF, was used as the F^−^ source). Correspondingly, the appearance of a 450 nm emission band upon 285 nm excitation was observed for Fmoc‐FuF DMSO solution supplemented with F^−^ solution, but not with other anions (Figure 3c). In contrast, when unmodified Fmoc‐FF was tested for interaction with F^−^, no change in the emission spectrum was detected (Figure 3c). The sensitivity of the Fmoc‐FuF optical response to F^−^ was quantified by measuring the fluorescence during gradual addition of aqueous F^−^ solution into Fmoc‐FuF DMSO solution. The fluorescence intensity (FI) was found to increase in a concentration‐dependent manner (Figure 3d). Examining the FI at 450 nm versus equivalents (equiv.) of F^−^ showed that a significant increase of the intensity is observed in the range of 0–20 F^−^ equiv. (Figure S6a,b, Supporting Information). The linear fitting of the plot log(FI) versus log[F^−^] in the range of 1–10 equiv. (*R* ^2^ = 0.99; Figure S6c, Supporting Information) and the corresponding plot of FI versus concentration (Figure 3e) indicate that Fmoc‐FuF can be used to reliably determine F^−^ concentration in the range of (4.93–50) × 10^−6^ m. The lower limit value of 4.93 × 10^−6^ m is similar to that of previously reported fluoride sensors.^42^, ^43^ The observed interaction between Fmoc‐FuF and F^−^ was confirmed using ^1^H NMR, which showed that both NH resonances (NH1 = 6.74 ppm; NH2 = 5.98 ppm; Figure S6d, Supporting Information) shifted significantly after the addition of F^−^, in line with a previous report.^44^ These data indicate that the F^−^ interacts predominantly with NH protons of the urea moiety via hydrogen bonding. The response of Fmoc‐FuF to F^−^ also extends to the mechanical properties of the hydrogel. A partial gel–sol transition was induced upon the addition of F^−^ to a preformed Fmoc‐FuF hydrogel, presumably due to the disruption of hydrogen bonds^45^ (Figure 3f). This mechanical response was investigated by measuring *G*′ of Fmoc‐FuF hydrogels (0.5 wt%) prepared with different equiv. of F^−^ (while maintaining the final gel volume fixed). Frequency sweep measurements demonstrated that *G*′ decreased with increasing concentrations of F^−^, reaching a value of 100 Pa in the presence of 6.0 F^−^ equiv. (Figure 3g). TEM analysis of this quasiliquid (Figure 3h; Figure S6e, Supporting Information) confirmed the breakdown of the fiber network into very short fibrils and amorphous matter. In contrast, the Fmoc‐FF hydrogel did not interact with F^−^, as it remained intact in a vial inversion test following overnight incubation with a 6.0 equiv. of F^−^ solution (Figure S6f, Supporting Information). We note that neither Fmoc‐FuF nor Fmoc‐FF hydrogels dissolved when sodium phosphate buffer, of the same pH and concentration as the TBAF solution (100 × 10^−3^ m, pH ≈ 7), was added to preformed hydrogels instead of TBAF (Figure S6f, Supporting Information). It is therefore concluded that, in contrast to the Fmoc‐FF hydrogel, the Fmoc‐FuF hydrogel is mechanically impaired by interaction with F^−^.

**Figure 3:**
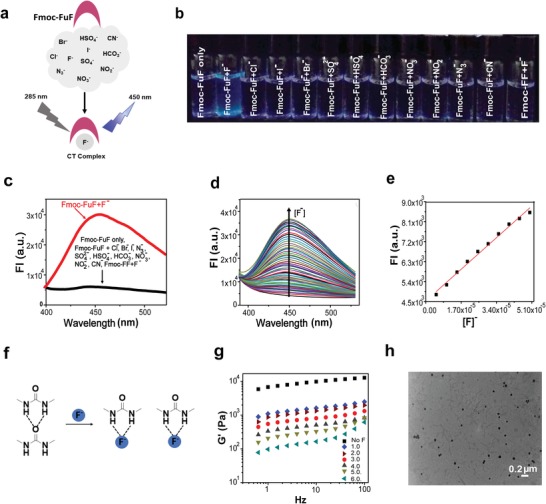
Selective anion binding by Fmoc‐FuF. a) Schematic representation of selective binding of F^−^ by Fmoc‐FuF, forming a charge‐transfer complex. b) Photograph of Fmoc‐FuF (5 × 10^−6^ m) DMSO solutions after the addition of 500 equiv. of various aqueous anion solutions under UV illumination (λ_ex_ = 365 nm). c) Fluorescence emission spectra of Fmoc‐FuF (5 × 10^−6^ m) DMSO solutions upon the addition of 450 equiv. of various aqueous anion solutions (λ_ex_ = 285 nm). d) Fluorescence intensity (FI) of Fmoc‐FuF (5 × 10^−6^ m) in DMSO solutions upon titration with 0–1000 equiv. of aqueous F^−^ solution (λ_ex_ = 285 nm). e) Plot of log[FI] versus F^−^ concentration. Red line is a linear fit, *R* ^2^ = 0.99. f) Schematic illustration of breakage of Fmoc‐FuF hydogel by F^−^. Dashed lines represent hydrogen bonds. g) Frequency sweep measurement of Fmoc‐FuF hydrogels (0.5 wt%) containing increasing F^−^ concentration (0–6.0 equiv. of F^−^). h) TEM image of Fmoc‐FuF gel (0.5 wt%) containing 6.0 equiv. of F^−^.

The next functionality tested was the ability of the Fmoc‐FuF hydrogel to reduce metal ions into nanoparticles in situ. We selected to synthesize Au and Ag nanoparticles (AuNPs and AgNPs) in order to demonstrate gel‐mediated nanoparticle synthesis^46^, ^47^, ^48^ and to test the involvement of the urea group, which was previously associated with metal nanoparticle synthesis.^49^, ^50^, ^51^ Nanoparticle‐containing Fmoc‐FuF hydrogels were prepared by gelating either 5 × 10^−3^ m HAuCl_4_ or 10 × 10^−3^ m AgNO_3_ aqueous solutions (with DMSO at 20% (v/v)) and allowing the nanoparticles to form in the dark in situ. This process resulted in color change to dark pink within 4 h in the former case or dark gray after 3 days in the latter (Figure S7a,b, Supporting Information). Hydrogels containing AuNPs or AgNPs showed characteristic surface plasmon resonance (SPR) bands at 530 and 430 nm, respectively (**Figure** **4**a). TEM imaging showed that in either case, nanoparticle formation did not alter the underlying fibrous morphology of the gel. Instead, nanoparticles appeared to aggregate along Fmoc‐FuF fibers (Figure 4b,c). The presence of AuNPs or AgNPs on the fibers was confirmed by selective area electron diffraction (SAED; Figure S7c,d, Supporting Information) and energy‐dispersive X‐ray (EDX) spectroscopy (Figure S7e,f, Supporting Information). Notably, no external reducing agent was added, nor was any external activation applied, such as heat, light, or pH modulation. In striking contrast, Fmoc‐FF hydrogel of the same concentration and DMSO content failed to reduce these metal ions into nanoparticles even after prolonged co‐incubation (Figure S7g, Supporting Information). These data suggest that the backbone urea group either directly reduces metal ions or facilitates metal ion reduction by the solvent, and thus allows for the Fmoc‐FuF gel as a whole to act as a template for in situ synthesis of AuNPs and AgNPs. Metal nanoparticles are commonly used as catalysts in various chemical reactions. The catalytic function of the AuNP‐containing Fmoc‐FuF xerogel was tested using a model reaction, in which 4‐nitrophenol (4‐NP) is reduced to 4‐aminophenol (4‐AP).^52^, ^53^ Time‐dependent UV–vis spectroscopy showed the characteristic decrease in intensity of the 400 nm band and the concomitant increase in absorption around 300 nm, associated with the conversion of 4‐NP to 4‐AP (Figure 4d). The rate constant of the reaction calculated from the slope of the plot of log(*A*) versus time was 0.089 min^−1^ (Figure S8a, Supporting Information). Importantly, reusing the AuNP‐containing xerogel as a catalyst was possible following washing with water and drying, with the reaction constant remaining nearly identical throughout a total of four reaction cycles (Figure 4e; Figure S8b, Supporting Information).

**Figure 4:**
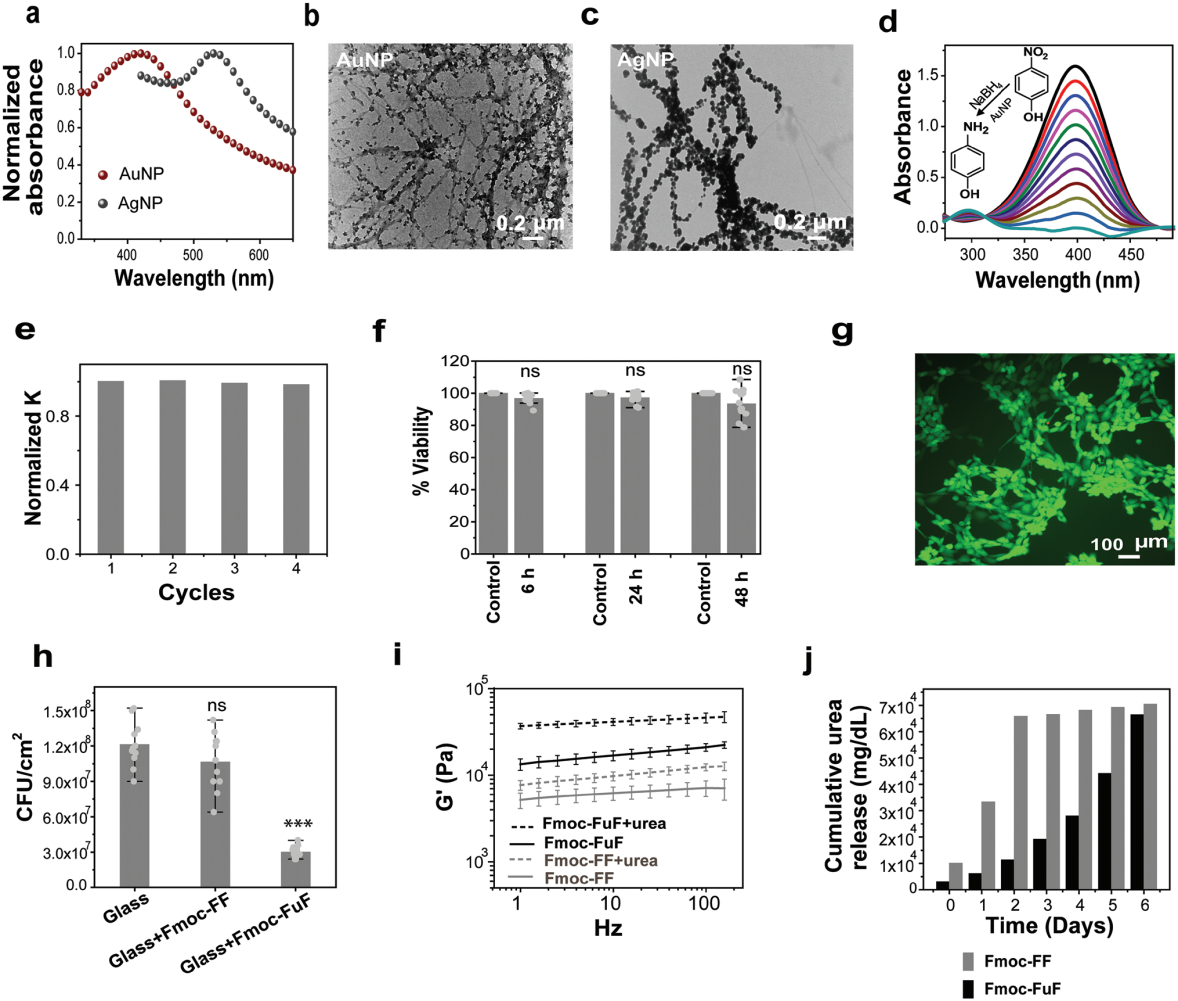
Diverse functionalities of the Fmoc‐FuF hydrogel. a) UV–visible spectra showing SPR bands of AuNPs or AgNPs formed in situ in the hydrogel (hydrogels were diluted threefold prior to measurement). b,c) TEM images of b) AuNP‐ and c) AgNP‐decorated fibers. d) Time‐dependent UV–vis spectra and e) corresponding normalized rate constant for repeated reaction cycles of the reduction of 4‐NP to 4‐AP, catalyzed by AuNP‐containing Fmoc‐FuF xerogel. f) Cell viability as determined by XTT assay, performed at different timepoints on 3T3 fibroblast cells cultured in naïve medium (control) or medium preincubated with Fmoc‐FuF hydrogel. Data represent mean ± SD from three independent experiments. Each timepoint was compared to control using Student's *t*‐test, *P* > 0.05 for all comparisons, indicating no statistically significant differences (ns). g) Representative Live/Dead staining of 3T3 fibroblast cells after 48 h of incubation on an Fmoc‐FuF hydrogel scaffold, showing a high abundance of living cells (green) and the negligible presence of dead cells (red). h) Bacterial adhesion to bare glass and glass surfaces coated with either Fmoc‐FF or Fmoc‐FuF xerogels, as per CFUs' count. Difference between Fmoc‐FuF coating and bare glass is significant (*P* < 0.001), difference between Fmoc‐FF coating and bare glass is not significant (ns, *P* > 0.05), as per Student's *t*‐test. Data represent mean ± SD from three independent experiments. i) Comparison of *G*′ values of Fmoc‐FuF and Fmoc‐FF hydrogels with or without encapsulated urea. Data represent mean ± SD (*n* = 3 hydrogels per condition). j) Comparison of cumulative urea release from urea‐containing Fmoc‐FuF (black) or Fmoc‐FF (gray) hydrogels, measured over a period of 6 days.

We next explored functionalities of the Fmoc‐FuF hydrogel related to interaction with mammalian and bacterial cells in order to evaluate its potential use in biomedical as well as antifouling applications. Compatibility with mammalian cells was tested using murine embryo fibroblasts (3T3). Initially, cells were incubated for 48 h with culture media pretreated by overnight incubation with Fmoc‐FuF hydrogels, and their viability was examined using 2,3‐bis‐(2‐methoxy‐4‐nitro‐5‐sulphophenyl)‐2*H*‐tetrazolium‐5‐carboxanilide (XTT) cell viability assay. Treated cells showed over 90% viability compared with control cells grown in naïve culture medium (Figure 4f), indicating that toxic molecules are not released from the hydrogel. Cellular compatibility was further tested by assessing the adhesion of cells to the hydrogel, a key requirement for biomedical applications. To this end, Fmoc‐FuF scaffolds were cultured with 3T3 cells and tested by Live/Dead analysis in situ, 48 h following seeding. Living cells were abundantly found on the gel scaffold (Figure 4g; control condition is shown in Figure S9, Supporting Information), showing that the biocompatibility of the Fmoc‐FuF hydrogel is similar to that of Fmoc‐FF,^15^ and in line with the reported biocompatibility of nonpeptidic urea–modified supramolecular hydrogels^29^ and covalent polymeric materials.^51^, ^52^ Hence, the Fmoc‐FuF hydrogel can support the attachment of cells, as required for biomedical applications.^54^, ^55^ Interestingly, whereas mammalian cell adhesion was supported by the hydrogel, bacterial adhesion (but not viability) was inhibited by its xerogel coating. Considering the application of poly(urea)‐based substrates as antifouling coatings,^56^ we examined similar functionality for the Fmoc‐FuF xerogel. Inhibition of bacterial adhesion was examined by a fouling test, in which glass surfaces, either bare, coated with Fmoc‐FuF, or coated with Fmoc‐FF, were incubated in *Escherichia coli* cultures at 37 °C overnight. Following removal of the surfaces from the culture, adhered bacteria were detached and plated, and the number of colony‐forming units (CFUs) was subsequently counted. Decent reduction in bacterial adhesion was achieved by Fmoc‐FuF xerogel coating, where 3 × 10^7^ CFUs cm^−2^ were counted as compared with 1.2 × 10^8^ CFUs cm^−2^ for bare glass, representing 0.6 log reduction. In contrast, for Fmoc‐FF xerogel coating 1.1 × 10^8^ CFUs cm^−2^ were counted, indicating the absence of an antifouling effect by Fmoc‐FF (Figure 4h). Furthermore, as a hydrogel, Fmoc‐FuF did not present antibacterial activity as per quantitative growth analyses of hydrogel‐grown bacteria and bacterial Live/Dead carried out on planktonic bacteria incubated with the hydrogels (Figure S10, Supporting Information), whereas Fmoc‐FF did present such activity (Figure S10, Supporting Information), as reported.^57^ Taken together, these results show that the urea backbone modification of Fmoc‐FuF ultimately promotes antifouling activity, albeit to a limited extent. Importantly, such activity was not demonstrated by Fmoc‐FF.

Finally, considering its inherent urea modification, we tested if Fmoc‐FuF could encapsulate free urea in the gel matrix for subsequent slow release. Slow release of the nitrogen source urea is desirable in agricultural fertilizers, where various strategies have been applied to delay urea release from fertilizers in order to maintain a steady supply of nitrogen for crop growth as well as reduce environmental pollution by urea leakage into the soil.^58^, ^59^ Fmoc‐FuF was found to gelate urea solutions such that a final concentration of up to 8 m urea in the gel was obtained. These urea‐containing gels were characterized and their ability to release urea was evaluated. Compared to typical Fmoc‐FuF hydrogel, the urea‐containing Fmoc‐FuF gel showed a denser network of fibers (Figure S11, Supporting Information) and a marked increase of 2.5‐fold in its *G*′ value (at 10 Hz; Figure 4i). Interestingly, inclusion of urea in an Fmoc‐FF gel increased its *G*′ value but only by less than 1.5‐fold (at 10 Hz), such that it was lower than the *G*′ value of the urea‐containing Fmoc‐FuF gel by more than an order of magnitude (Figure 4i). Notably, *G*′ value increased for both Fmoc‐FuF and Fmoc‐FF gels, despite^19^ the increase in gel pH (5.5 ± 0.2 for Fmoc‐FF and 5.6 ± 0.3 for Fmoc‐FuF). These data indicate that encapsulated urea overall contributes to *G*′, but to a greater extent for Fmoc‐FuF. We attribute this phenomenon to increased hydrogen bonding, which is expected to be more dominant for the urea‐modified^31^ Fmoc‐FuF. The release of urea from the urea‐containing Fmoc‐FuF gel was evaluated next. To this end, gels were immersed in water for a period of 6 days, during which the release of urea into the aqueous environment was monitored daily by a colorimetric assay.^60^ The concentration of released urea increased gradually over time, from 4% (day 1) to 91% (day 6) of its initial concentration in the gel (Figure 4j). In contrast, urea release from urea‐containing Fmoc‐FF gel was considerably more rapid, with 91% of the initial concentration in the gel released over a period of 2 days (Figure 4j). Hence, Fmoc‐FuF, but not Fmoc‐FF, may be utilized for encapsulation and slow release of urea. Gelation of urea by Fmoc‐FuF apparently allows for similar or improved urea release behavior as compared with other encapsulation methods,^61^, ^62^ although a comparative study will be required to fully assess this. Furthermore, as the use of existing slow‐release fertilizers is typically limited by their relatively non‐biodegradable, toxic coating materials,^63^, ^64^ the biocompatible Fmoc‐FuF hydrogel may provide a more ecofriendly means for achieving slow release of encapsulated urea in agriculture.

In summary, by incorporating a urea backbone modification in an extensively characterized peptide hydrogelator, we have obtained a novel peptidomimetic that gives rise to a multifunctional, stimulus‐responsive, and biocompatible supramolecular hydrogel. This hydrogel forms by self‐assembly of Fmoc‐FuF molecules into spheres and worm‐like irregular clusters, which then fuse to give rise to gel‐like fibers in a process mediated by aromatic stacking and hydrogen bonding. Fmoc‐FuF formed a hydrogel of high mechanical rigidity, with shear thinning and self‐healing properties. This hydrogel possesses diverse functional properties, namely anion sensing, metal‐ion reduction, mammalian cell scaffolding, antifouling capabilities, and the ability to slowly release encapsulated urea. Excluding cell scaffolding, these functional properties clearly stem from, or become significantly enhanced by, a urea backbone modification, as evident by direct comparison with the unmodified peptide hydrogelator Fmoc‐FF. This work demonstrates the significant impact of simple amide bond modification, thus laying the basis for improved biomaterial design toward practical applications.

## Conflict of Interest

The authors declare no conflict of interest.

## Supporting information

Supplementary
