# Supplementary material for: Expanding the Functional Scope of the Fmoc‐Diphenylalanine Hydrogelator by Introducing a Rigidifying and Chemically Active Urea Backbone Modification
Source: Adv Sci (Weinh). 2019 Apr 19;6(12):1900218. doi: 10.1002/advs.201900218 (PMC6619482; doi:10.1002/advs.201900218)
Supplement: Supplementary file 1 — Supplementary [file ADVS-6-1900218-s001.pdf]

Copyright WILEY-VCH Verlag GmbH & Co. KGaA, 69469 Weinheim, Germany, 2019.

ADVANCED SCIENCE
Open Access

Supporting Information

for *Adv. Sci.*, DOI: 10.1002/advs.201900218

Expanding the Functional Scope of the Fmoc-Diphenylalanine Hydrogelator by Introducing a Rigidifying and Chemically Active Urea Backbone Modification

Vasantha Basavalingappa, Tom Guterman, Yiming Tang, Sivan Nir, Jiangtao Lei, Priyadarshi Chakraborty, Lee Schnaider, Meital Reches, Guanghong Wei, and Ehud Gazit**

DOI: 10.1002/(advs.201900218)
Article type: Communication

Copyright WILEY-VCH Verlag GmbH & Co. KGaA, 69469 Weinheim, Germany, 2019.

Supporting Information

Expanding the Functional Scope of the Fmoc-diphenylalanine Hydrogelator by Introducing a Rigidifying and Chemically Active Urea Backbone Modification

Vasantha Basavalingappa, Tom Guterman, Yiming Tang, Sivan Nir, Jiangtao Lei, Priyadarshi Chakraborty, Lee Schnaider, Meital Reches, Guanghong Wei, Ehud Gazit**

Experimental Section

Materials: Fmoc-FuF was purchased from DG peptides (Hangzhou, China). Dimethyl sulfoxide (DMSO), metal ions, *4*-nitro phenol, tetrabutylammonium salts (TBAX, X = (Cl, Br, F, I, CN)), $NaHSO_4$, $NaBH_4$ were purchased from Sigma-Aldrich (Rehovot, Israel). Urea and $NaHCO_3$ were purchased from Merck (Darmstadt, Germany). Na_2SO_4 was purchased from Acros Organics (Morris plains, NJ, USA), $NaNO_3$, $NaNO_2$, NaN_3 were purchased from Frutarom (Haifa, Israel) and ultrapure water from Biological Industries (Beit Haemeck, Israel). QuantiChrom Urea Assay Kit was purchased from BioAssay Systems (Hayward, CA, USA). 100 mM Sodium phosphate buffer, pH 7.0 was purchased from Sigma-Aldrich (Rehovot, Israel).

Gel Preparation: Fmoc-FuF and Fmoc-FF stock solutions were prepared by dissolving the peptides in DMSO at a concentration of 25 mg/mL. The hydrogels were prepared by diluting either stock solution in Milli-Q water to a final peptide concentration of 5 mg mL^{-1} (0.5 wt%) and 20% (v/v) DMSO. For the preparation of urea-containing gels, DMSO stock solution was diluted with an aqueous 10 M urea solution to obtain final urea concentration of 8 M, 0.5 wt% gelator, and 20% (v/v) DMSO. Metal salts were encapsulated by diluting DMSO gelator stock with aqueous 100 mM metal ion solution to obtain final metal ion concentration of 5 mM

(Au) and 10mM (Ag). Hydrogels with different F^- concentrations were prepared by diluting DMSO stock solution with 100 mM tetrabutylammonium fluoride (TBAF) as specified.

Anion Binding: The emission spectra of the gel and anion binding of the monomer were recorded using a Horiba JobinYvon FL3-11 fluorimeter (Horiba Jobin Yvon, Edison, NJ, USA). A quartz cuvette with an optical path length of 1 cm (Hellma Analytics, Müllheim, Germany) was used. The experiments were carried out by setting the excitation wavelength to 285 nm and recording the emission between 300-500 nm, with excitation and emission slits of 5 nm. Photograph of fluorescence response was obtained by illuminating with VL-6.LC UV lamp (Vilber, Eberhardzell, Germany).

NMR Spectroscopy: ^{1}H NMR was recorded by dissolving 5 mg of Fmoc-FuF in 500 µL of DMSO-d_6 (solution state) and 0.5 wt% gel with a DMSO-d_6-H_2O at a ratio of 20% (v/v) (gel state).^{1}H NMR spectra were recorded at 25 ºC on Bruker 500 MHz spectrometer. Interactions of the gelator with F^-, using TBAF as the F^- source, were examined by ^{1}H NMR in DMSO-d_6. In this case, the initial concentration of Fmoc-FuF in DMSO-d_6 was 20 mM, while the F^- solution was prepared at a 10-fold higher concentration in the same solvent. The guest solution was typically added in 10 µl aliquots, representing 0.1 equivalents of the guest with respect to the host. Spectra were recorded after each addition and the trace followed. Chemical shifts were expressed as parts per million and calibrated against TMS as an external reference. Water signal suppression was carried out by excitation sculpting techniques using gradients.[1] NMR data were processed and analysed using Topspin Version 3.5.

Chemical Catalysis: The reduction of 4-nitrophenol (4-NP) to 4-aminophenol (4-AP) was monitored using T60 UV-Vis spectrophotometer (PG Instruments, Leicestershire, UK). A rectangular quartz cuvette with an optical path length of 1 cm (Hellma Analytics, Müllheim, Germany) was used. Baseline-corrected UV-Vis spectra in the range of 200-500 nm were acquired. 200 µL of 1 mM 4-NP and 320 uL of 56 mM $NaBH_4$ was diluted with Milli-Q water to a final volume of 2 mL in a quartz cuvette, and 1 mg of the AuNPs-containing Fmoc-FuF

xerogel was then added. UV-Vis spectra following the catalytic reaction of 4-NP were immediately recorded at 1 min intervals. The reaction follows first order kinetics,[2] ln(A) *vs.* time = k, where, A = A_t/A_o, $A_{(t)}$ and $A_{(0)}$ are the absorption intensities of 4-AP at 300 nm at time t and zero, respectively, and k is the rate of the reaction calculated from the slope of the plot ln(A) *vs.* time.

UV-Vis spectra of AuNPs (formed by reduction of 5 mM of $HAuCl_4$ aqueous solution by 200 uL of 0.5 wt% gel) and AgNPs (formed by reduction of 10 mM of $AgNO_3$ aqueous solution by 200 uL of 0.5 wt% gel) were recorded using Biotek Synergy HT plate reader (Winooski, VT, USA) in a 96-well flat bottom Costar® plate (Corning Inc., New York, USA).

Urea Release: Hydrogels of Fmoc-FuF or Fmoc-FF, with or without urea (see *Gel Preparation*), were prepared at 1.5 mL volume in a 6-well plate. The gels were allowed to form for 24 h in the plate and then 6 mL of water was added on top of each gel. For each hydrogel, 5 µL of the immersing water were immediately transferred into a 96-well flat bottom Costar® plate (Corning Inc.) for detection of urea at timepoint zero using 200 µL urea assay reagent (BioAssay Systems). Absorbance was recorded at 520 nm after 20 min of equilibration (according to the manufacturer’s instructions), using a Biotek Synergy HT plate reader. Finally, 5 µl of fresh water were added to maintain the initial volume. The experiment was performed for 6 days by daily sampling in the same manner. The concentration of urea released was calculated using equation 1:

$$\left(\text{Amount of urea released}\left(\frac{\text{mg}}{\text{dL}}\right) = \frac{OD_{sample} - OD_{blank}}{(OD_{standard} - OD_{blank})} * n * [STD]\right) \quad (1)$$

where OD_{sample}, OD_{blank} and $OD_{standard}$ are optical density (OD) values of sample, blank (water and Fmoc-FuF/Fmoc-FF gel without urea) and standard, respectively. n is the dilution factor. [STD] = 50, urea standard concentration in mg/dL. The calculated concertation of released urea was plotted against time (days) to obtain cumulative urea release in mg/dL.

Transmission Electron Microscopy (TEM): A piece of Fmoc-FuF gel was placed on a 400-mesh copper grid covered by a carbon-stabilized Formvar film (Electron Microscopy Science, Fort Washington, PA, USA). After 3 min, excess fluid was blotted off. The samples were dried under ambient conditions and the micrographs were recorded using a JEM-1400Plus Transmission Electron Microscope operating at 80 kV (JEOL, Tokyo, Japan).

Imaging and selective area electron diffraction (SAED) was performed using a Tecnai F20 high-resolution field-emission transmission electron microscope (Philips, Eindhoven, the Netherlands) operating at 200 kV. Data analysis was performed using the Gatan Microscopy Suite 3 software package. Energy dispersive X-ray (EDX) analysis was performed using the attached EDAX detector.

High-Resolution Scanning Electron Microscopy (HRSEM): A piece of freshly formed Fmoc-FuF gel was placed on a microscope glass cover slip and allowed to dry under ambient conditions. Images were recorded using a JSM-6700F FE-SEM (JEOL) operating at 2 kV after coating the sample with Cr.

Atomic Force Microscopy (AFM): AFM imaging was performed by depositing 10 μl of gel onto freshly cleaved V1 grade mica (Ted Pella, Redding, CA, USA). The samples were allowed to dry under ambient conditions. Images were obtained using AIST-NT Smart AFM system in non-contact (tapping) mode using 100 mm long silicon nitride cantilevers (OMCL-RC800PSA-W, Olympus, Tokyo, Japan) with resonance frequency of 70 kHz. Images were visualized and analysed using the WSxM imaging software[3] (Nanotec Electronica S.L., Madrid, Spain).

Rheology: All rheological measurements were conducted on gels prepared at 0.5 wt% and 300 μL volume, prepared individually in 2.5 mL syringes (Pic Solution, Artsana S.p.A, Grandate, Italy). For each syringe, the tip was removed from the barrel beforehand by cutting, so as to obtain a wide opening (at the diameter of the barrel) at the expense of the tip. Gels were prepared by adding the DMSO stock of the gelator into the syringe (while its plunger is pulled

back) and diluting with water as described in the Gel Preparation section. The solution was mixed inside the syringe by pipetting and the gels were allowed to form for 24 h inside the syringe prior to measurement. Mechanical properties were characterized by an ARES-G2 rheometer (TA Instruments, New Castle, DE, USA) using 20 mm parallel-plate geometry, by maintaining a gap size of 1 mm at room temperature. Gels were transferred from the syringe to the rheometer stage by gently pushing the plunger.

pH measurement: pH was measured for gels that were allowed to form for 24 h. FC200B pH probe (Hanna instruments, USA), with a 6 mm x 10 mm conical tip and 0.1 pH unit accuracy, was calibrated prior to measurement. Readings were taken 1 min after inserting the conical tip into a gel.

Turbidometry: 100 uL of 0.5 wt% gel samples were prepared in a 96-well flat bottom Griener UV star microplate (Greiner bio-one, North Carolina, USA). OD at 400 nm was measured every 20 s for 30 min using a Biotek Synergy HT plate reader (Winooski, VT, USA).

Antifouling Assay: E.coli (ATCC 25922) were grown overnight in tryptic soy broth (TSB) medium at 37 ˚C, in loosely capped tubes with agitation (120 rpm), to early stationary phase. Then, the bacteria were centrifuged and washed 3 times with PBS, re-suspended, and diluted to 10^7 CFU/mL with TSB. Glass slides, 1x1 cm^2 n size were coated with Fmoc-FuF or Fmoc-FF hydrogels and allowed to dry at ambient temperature. Coated or bare glass slides were placed horizontally in a 6-well plate. 4 mL of the bacterial culture were transferred to each well and the plate was incubated in a humidified incubator at 37 ˚C overnight. After incubation, the substrates were gently rinsed with 3 mL PBS and transferred into test tubes containing 3 mL PBS. The test tubes were then sonicated for 1 min to detach the bacteria from the substrates, and vortexed for 15 s. The number of viable bacteria was determined by plating the samples in 10-fold serial dilutions on lysogeny broth (LB) agar plates.[4]

Cell Viability: For 2,3-bis-(2-methoxy-4-nitro-5-sulphophenyl)-2*H*-tetrazolium-5-carboxanilide (XTT) assay, 3T3 mouse fibroblast cells were purchased from ATCC and cultured in Dulbecco's modified Eagle's medium (DMEM) supplemented with 10% fetal calf serum, 100 U mL^{-1} penicillin, 100 U mL^{-1} streptomycin, and 2 mM L-glutamine (all from Biological Industries, Beit Haemeck, Israel). Hydrogels were formed in a 24-well plate using ultrapure water and repeatedly washed with culture medium for 3 days. The gels were incubated with culture medium for 12 h and sterilized under UV. In parallel, cells were cultured in 96-well tissue microplates (100 µl per well) and allowed to adhere overnight at 37 °C. 100 µl of non-treated culture medium or medium pre-incubated with gel was added to each well. After incubation for 24 h at 37 °C, cell viability was evaluated using the XTT cell proliferation assay kit (Biological Industries, Beit Haemeck, Israel) according to the manufacturer's instructions. Briefly, 100 µL of the activation reagent was added to 5 ml of the XTT reagent, followed by the addition of 100 µl of activated XTT solution to each well. After 2.5 h of incubation at 37 °C, colour intensity was measured using ClarioStar plate reader (BMG Labtech, Ortenberg, Germany) by measuring the absorbance at 450 and 630 nm. Each experiment was repeated 3 times.

For Live/Dead assay, gels were formed in a 24-well plate and repeatedly washed for 3 days with culture medium and then sterilized by UV light. Subsequently, the pH of the final medium over the gels was measured to be 7.4-7.8. After reaching a confluence of 90%, the cells were separated from the Petri dish using trypsin A, and 60,000 cells in 100 µl of fresh culture medium were seeded per well on the prewashed gel samples followed by incubation for 48 h. A fluorescent Live/Dead staining assay (Sigma-Aldrich, Rehovot, Israel) containing fluorescein diacetate (6.6 µg/mL) and propidium iodide (5 µg/mL) was then performed according to the manufacturer's instructions. The labelled cells were immediately visualized using an Eclipse Ti-E Microscope (Nikon, Tokyo, Japan).

Antibacterial activity: Gels were formed in a 24-well plate and repeatedly washed for 3 days with milli-Q water and then sterilized by UV light. *E. coli* bacterial samples (ATCC 25922) at an OD_{600} of 0.1 were grown on each of the hydrogels for 18 h at 37 °C. The hydrogels were then washed thrice with LB. These washes were collected, centrifuged to pellet the bacteria in each sample, and washed again with sterile saline. Bacterial viability was then evaluated by Live/Dead bacterial viability assay using the L13152 LIVE/DEAD BacLight Bacterial Viability Kit (Molecular Probes, OR, USA), and by OD readings of each sample at 600 nm. Results displayed are representative of three independent experiments conducted.

Coarse-Grained Molecular Dynamics Simulations (CG-MD): Microsecond-long MD simulations on 200/400/600 Fmoc-FUF systems were carried out using the GROMACS package (version 2016.4)[5] in combination with the MARTINI coarse-grained (CG) model (version 2.2).[6] The mapping from all-atom model of an Fmoc-FuF molecule to CG model and the interaction types of the CG beads are shown in Figure S3. The trajectory analyses were performed using in-house-developed codes and tools implemented in the GROMACS package. SASA fraction of each group and the free energy landscape were used to analyse the simulation data.

Microsecond-long CG-MD simulations were performed on 200/400/600 Fmoc-FuF systems in aqueous solutions containing about 20% (v/v) DMSO. The Fmoc-FuF molecule can be divided into three groups: the fluorenyl ring, the two phenyl rings, and the polar main chain, which are coloured in orange, blue, and purple, respectively (Figure S5). Each Fmoc-FuF molecule was represented by 17 CG beads: four beads for the main chain, three for each phenyl ring and seven for fluorenyl groups. DMSO and water molecules were represented using Na and P4 interaction types of beads, respectively.

In the initial state of the three simulated systems (200/400/600 Fmoc-FuF), the Fmoc-FuF molecules were randomly placed in a 20×20×20 nm^3 cubic box containing 50452 water beads

and 12800 DMSO beads. Electrostatic interactions were treated using the Particle Mesh Ewald (PME) method,[7] with a real space cut-off of 1.4 nm. The vdW interactions were calculated using a cut-off of 1.4 nm. The solute and solvent were separately coupled to an external temperature bath using a velocity rescaling method[8] and a pressure bath using the Parrinello-Rahman method.[9] The temperature and pressure were maintained at 298 K and 1 bar, respectively. The neighbour-list was updated every 10 steps with a cut-off distance of 1.4 nm using a Verlet buffer.[10]

The SASA fraction of each group (fluorenyl group, phenyl group, and main chain group) was defined as the percentage of the SASA of that group relative to the SASA of all Fmoc-FuF molecules at each time point. The angle between two benzene rings refers to the angle between the normal vectors of the two rings. If the angle was larger than 90°, the supplementary angle was used as the angle between the two benzene rings. Two benzene rings were considered only if their centroid distance was within 0.8 nm. The two-dimensional free energy landscape was constructed using the relation –RTln[P(angle, centroid distance)], where P(angle, centroid distance) is the probability of a stacking pattern to have a certain value of angle and centroid distance. The data in the last 0.2 μs of CG-MD trajectories were used to construct the free energy landscape. Trajectory visualization and graphical structure analysis were performed using the PyMOL software suite.[11]

Supporting Information Figures

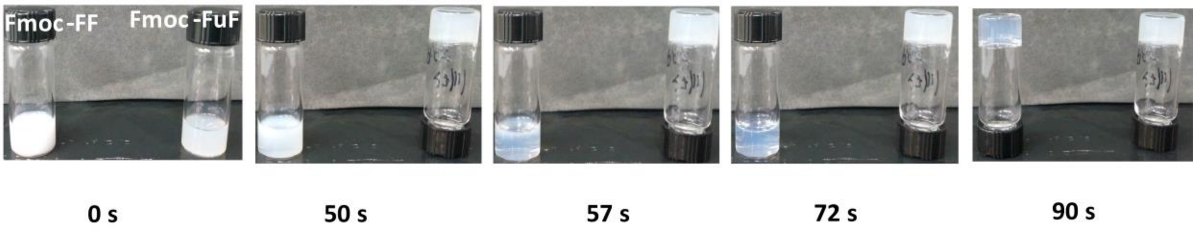

Figure S1. Photographs comparing gelation and optical clearance kinetics of Fmoc-FF and Fmoc-FuF.

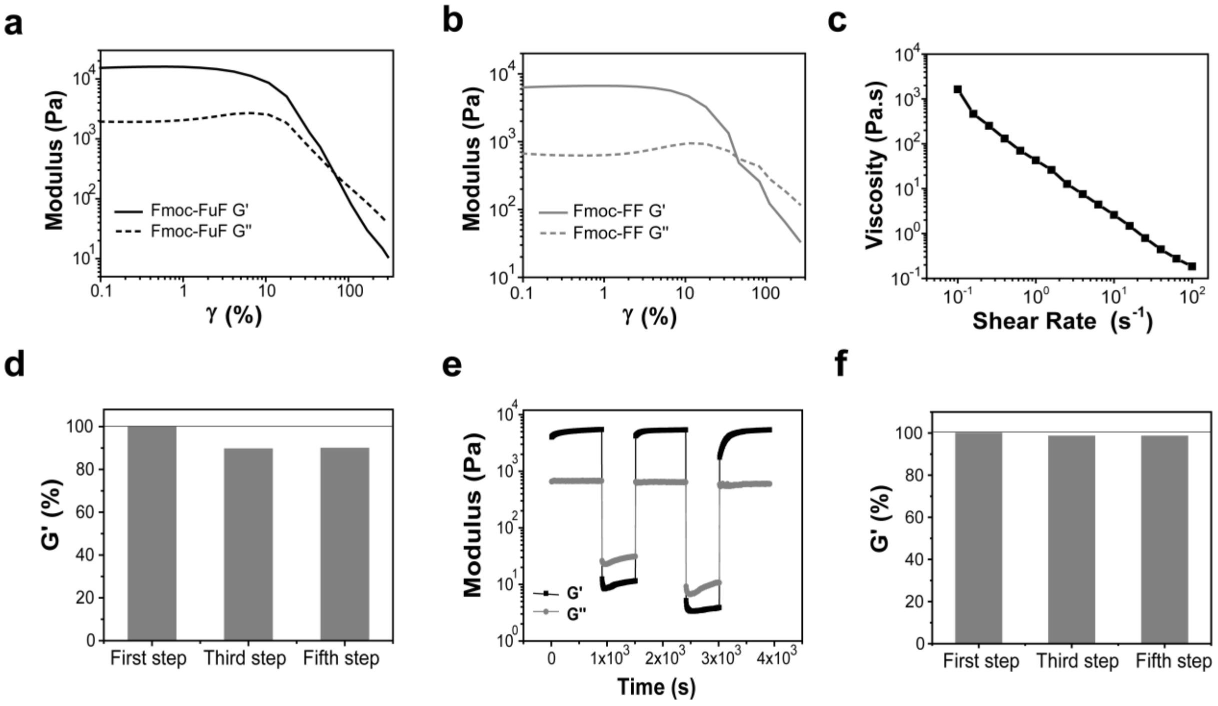

Figure S2. Hydrogel breakage strain value (γ) for a) Fmoc-FuF and b) Fmoc-FF. c) Flow sweep measurement showing shear thinning behaviour of Fmoc-FF hydrogel. d) Thixotropic behaviour of Fmoc-FuF hydrogel in a 5-step-loop time-sweep experiment, plotted as % reduction of G′ as compared with the first step. e) 5-step loop time sweep measurement showing the thixotropic nature of Fmoc-FF hydrogel. f) Thixotropic behaviour of Fmoc-FF hydrogel in a 5-step-loop time-sweep experiment, plotted as % reduction of G′ as compared with the first step.

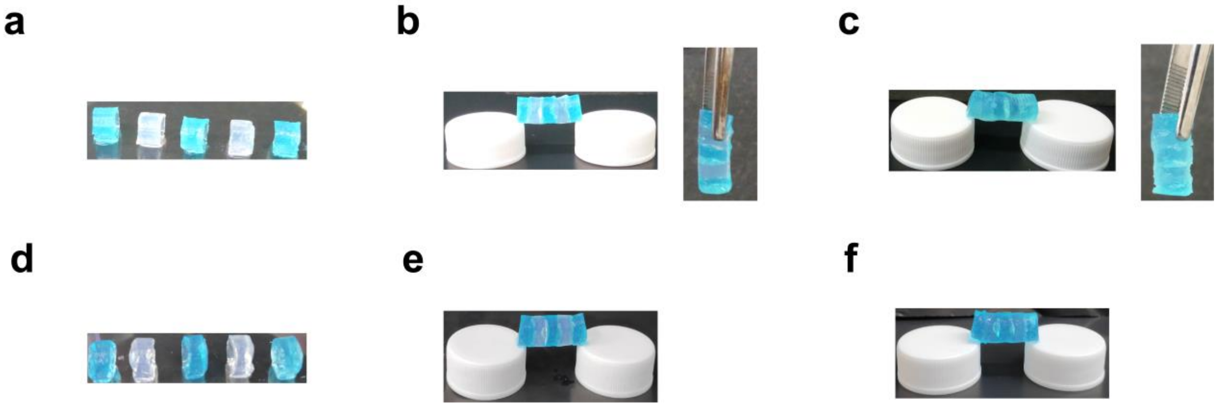

Figure S3. Self-healing of the Fmoc-FuF and Fmoc-FF hydrogels. a) Freshly cut hydrogels of Fmoc-FuF. b) Left: rejoined hydrogels, bridgeing a 2.5 cm-long elevated gap. Right: rejoined hydrogels, lifted vertically from the surface using forceps. c) Healed gel, similarly imaged after 2 h. d) Freshly cut hydrogels of Fmoc-FF. e) Rejoined hydrogels, bridgeing a 2.5 cm-long elevated gap. f) Healed gel, similarly imaged after 2 h. (Blue colored gel was obtained by the addition of methylene blue).

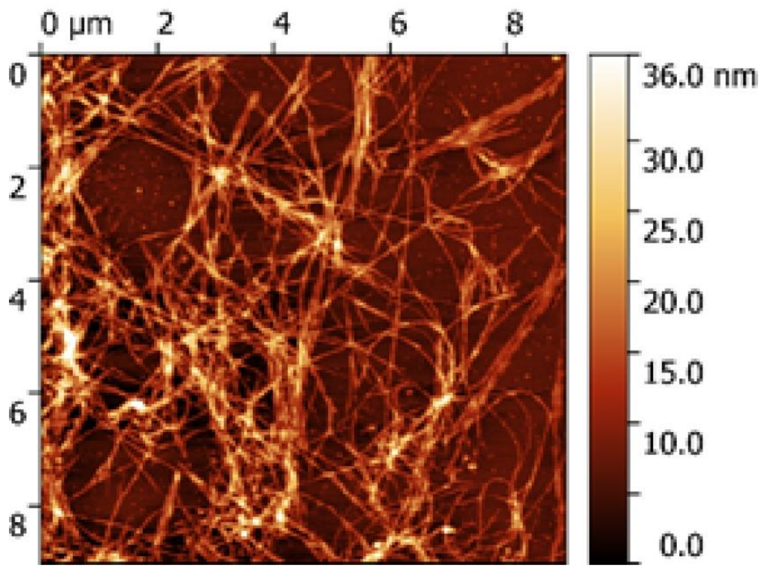

Figure S4. AFM image of Fmoc-FuF fibres.

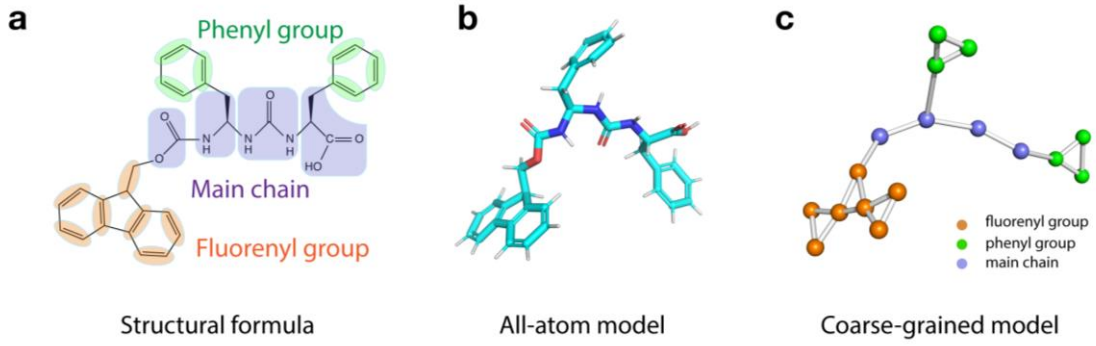

Figure S5. Structure of an Fmoc-FuF molecule. a) Chemical structure. b) All-atom model. c) Coarse-grained model.

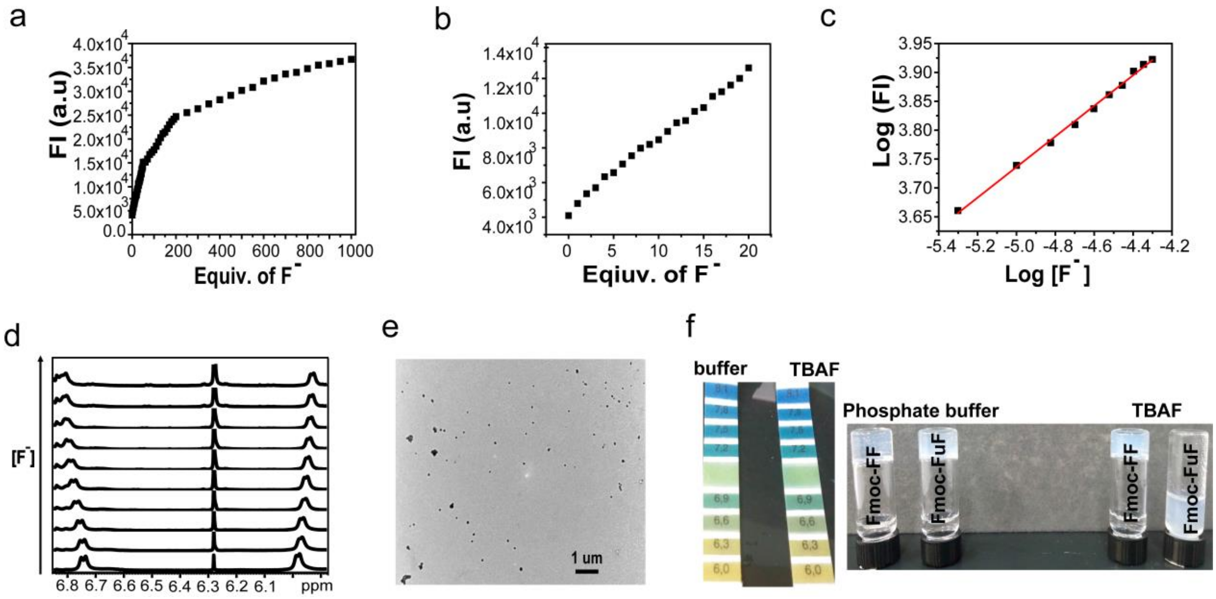

Figure S6. Binding of F^- by Fmoc-FuF. a) Fluorescence intensity (FI) *vs.* equiv. F^-. b) Enlarged view of the 0-20 equiv. range. c) Plot of log (FI) *vs.* log[F^-] in the range of 1-10 equiv. F^-. d) ^{1}H NMR spectrum of titration of Fmoc-FuF in DMSO (20 mM) against aliquots of TBAF in DMSO. e) TEM image of TBAF in DMSO-water. f) Left: photograph of pH test strip (0.3 unit resolution) after dipping in 100 uM phosphate buffer and 100 mM TBAF solution. Right: Photographs of vials containing Fmoc-FF and Fmoc-FuF hydrogels in 100 mM phosphate buffer (pH 7) or TBAF solution (pH ~7).

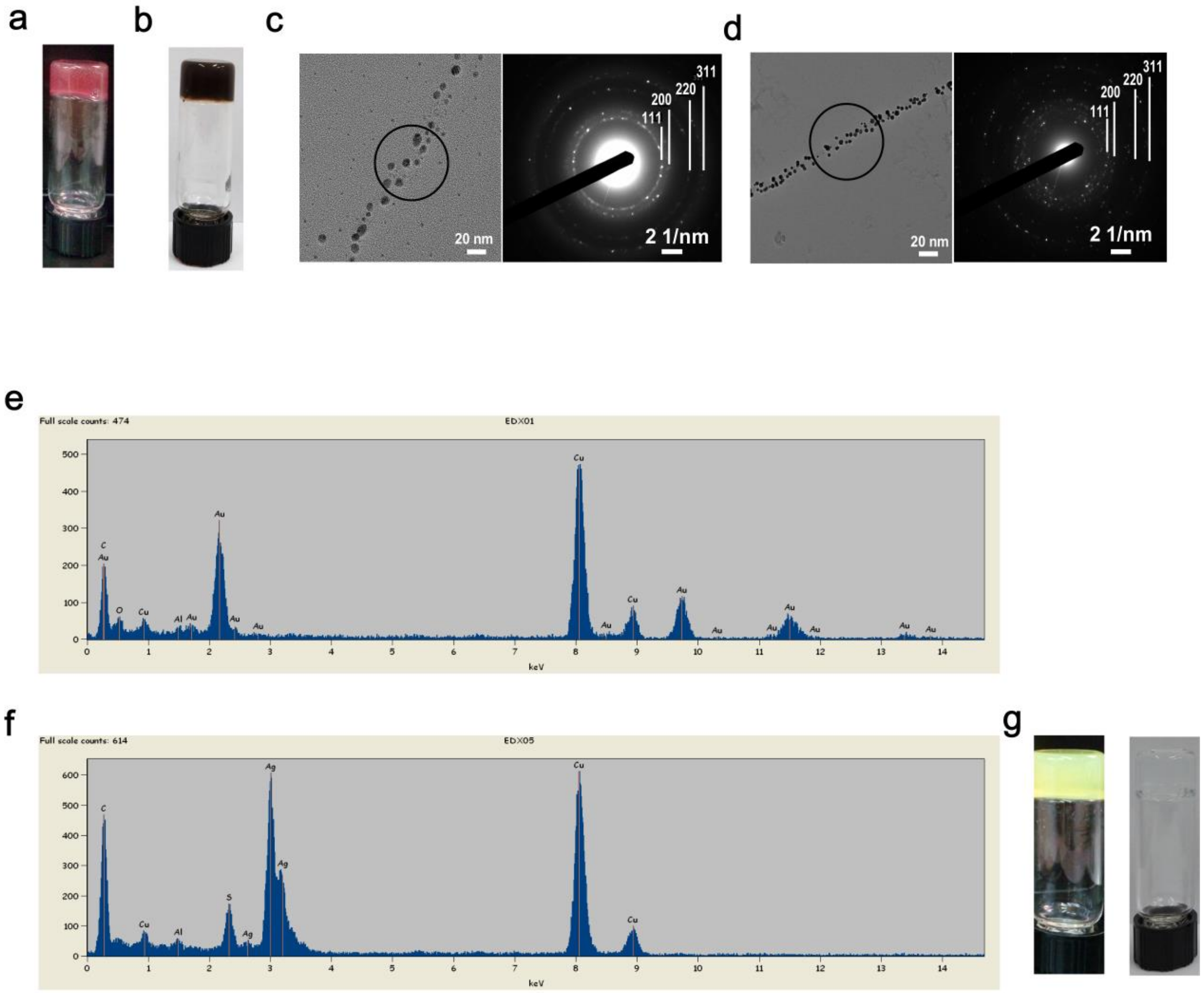

Figure S7. Metal ion reduction by the Fmoc-FuF hydrogel. a,b) Photographs of gels containing *in situ*-formed AuNPs (a) or AgNPs (b). c) Left: High resolution TEM (HRTEM) image of an AuNPs-decorated Fmoc-FuF fibre. Circle represents the area selected for electron diffraction. Right: corresponding diffractogram of AuNPs decorated fibre. d) Left: High resolution TEM (HRTEM) image of AgNPs-decorated Fmoc-FuF fibre. Circle represents the area selected for electron diffraction. Right: corresponding diffractogram of an AgNPs-decorated Fmoc-FuF fibre. For c-d, reflections are indexed to the 111, 200, 220, 311 planes of Au or Ag. e) EDX element analysis of AuNPs-decorated fibres. f) EDX elemental analysis of AgNPs-decorated fibres. For panels e, f, the presence of S, Cu and Al is due to residual DMSO, presence of TEM grid, and microscope inner beam reflection, respectively. g) Fmoc-FF hydrogel incubated with ionic Au or Ag. The colour change characteristic of AuNPs or AgNPs formation was not observed.

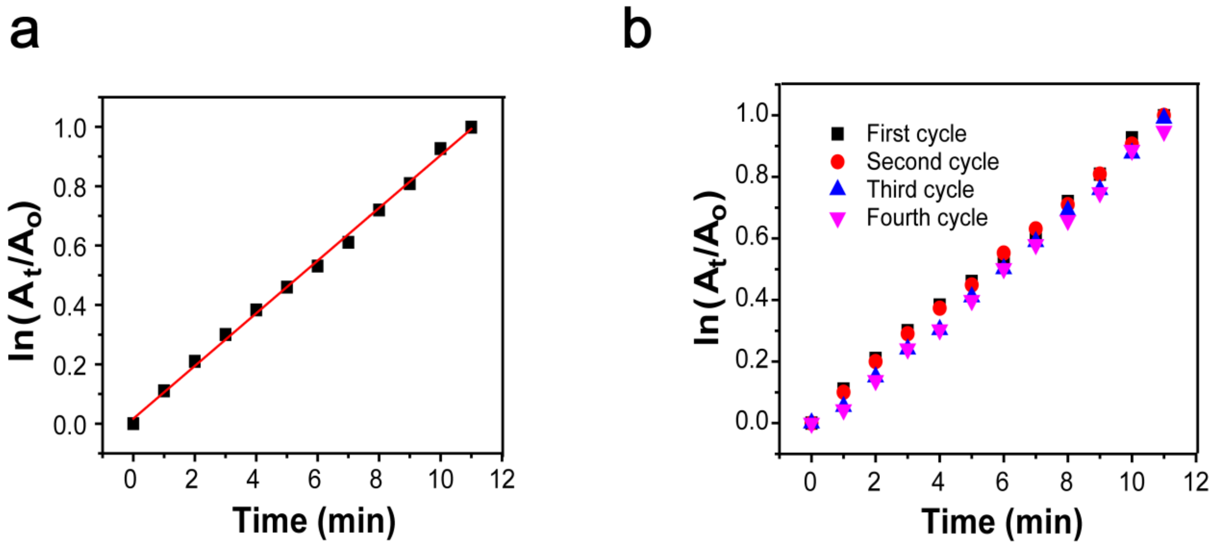

Figure S8. a) Plot of the absorbance at 300 nm *vs.* time for 4-NP reduction to 4-AP, catalysed by the AuNPs-containing Fmoc-FuF xerogel. Red line is a linear fit, R^2 = 0.99. b) Corresponding catalytic activity throughout four reaction cycles.

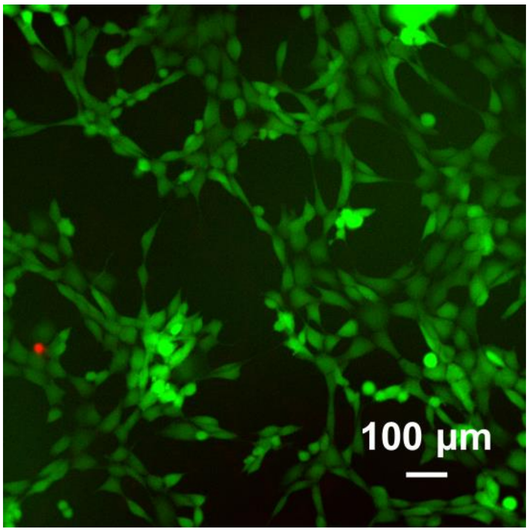

Figure S9. Representative Live/Dead staining from the control condition, showing 3T3 fibroblast cells after 48 h of incubation in a 24-well plate. The fluorescence image shows a high abundance of living cells (green) and the negligible presence of dead cells (red).

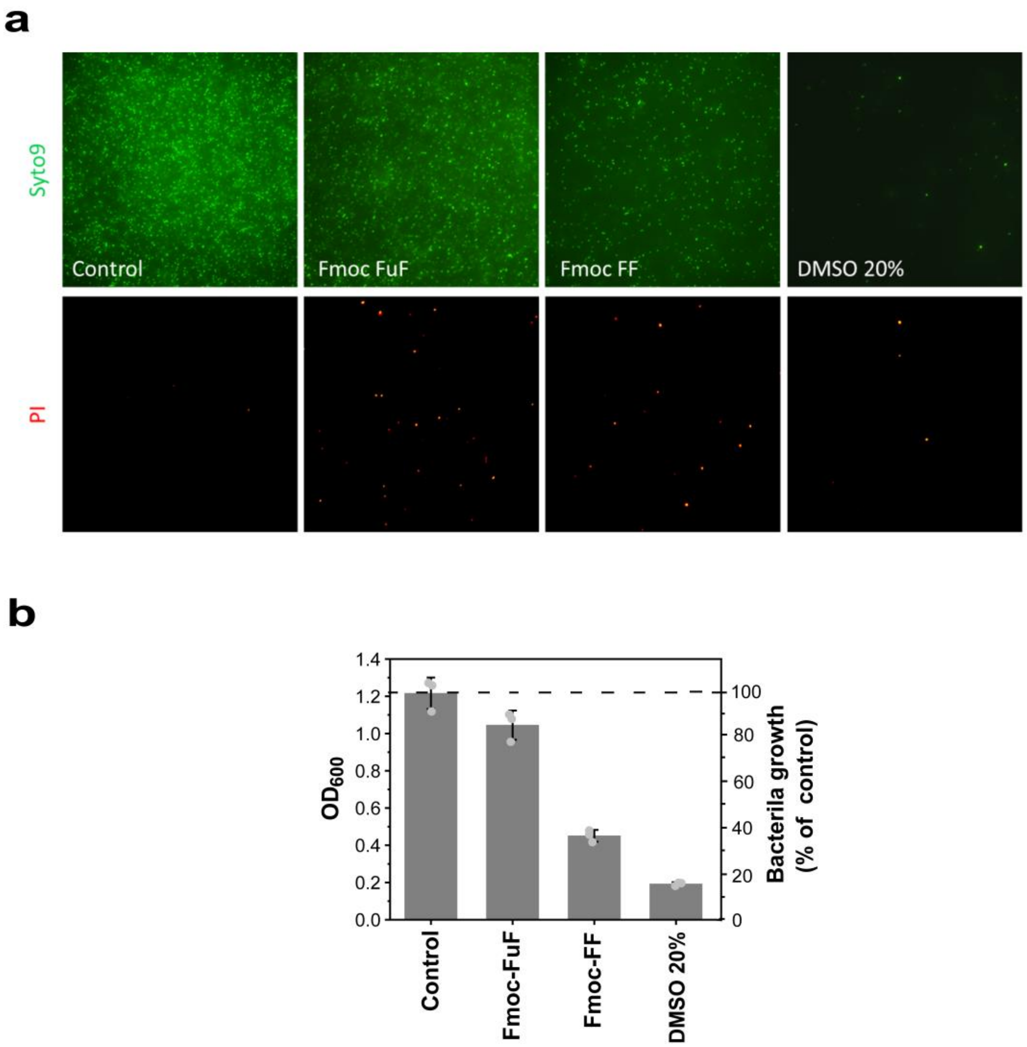

Figure S10. Evaluation of the antibacterial activity of Fmoc-FuF and Fmoc-FF hydrogels. a) Viability of *E.coli* bacteria as evaluated by bacterial Live/Dead assay. Green fluorescence of the Syto9 probe indicates bacterial cells with an intact membrane while red fluorescence of Propidium Iodide (PI) indicates dead bacterial cells. Data shows considerable reduction in viability for Fmoc-FF but not for Fmoc-FuF. b) Corresponding turbidometric growth analysis at 600 nm. Data represents mean ± SD, and shows significant ($P < 0.0001$) reduction in viabilty for Fmoc-FF but not significant (ns, $P > 0.05$) reduction for Fmoc-FuF, as compared with a naïve bacteria control by Student's *t*-test. For both panels, prior to analysis bacteria were grown for 18 h in LB medium on each of the hydrogels or in the presence of 20% (v/v) DMSO. The experiment was performed in triplicate. See Experimental Section for more details.

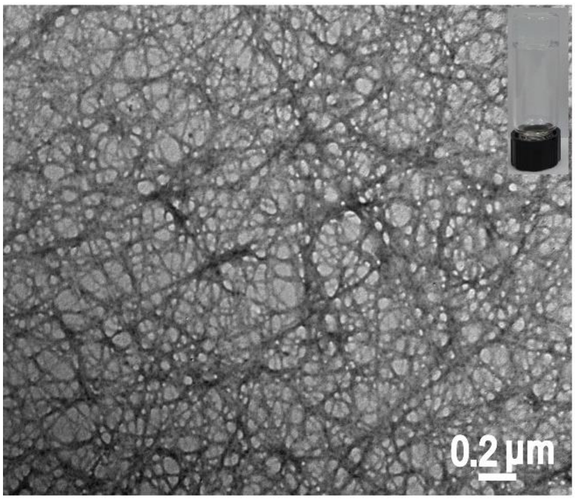

Figure S11. TEM image of urea-containing Fmoc-FuF hydrogel. Inset is a photograph of the hydrogel in an inverted vial.

References

[1] T. -L. Hwang, A. J. Shaka, *J. Magn. Reson. Series A*, **1995**, *112*, 275.

[2] G. Nyström, M. P. Fernández-Ronco, S. Bolisetty, M. Mazzotti, R. Mezzenga, *Adv. Mater.* **2016**, *28*, 472.

[3] I. Horcas, R. Fernández, J. M. Gómez-Rodríguez, J. Colchero, J. Gómez-Herrero, A. M. Baro, *Rev. Sci. Instrum*. **2007**, *78*, 013705.

[4] Gusnaniar, F. Hizal, C.-H. Choi, J. Sjollema, T. Nuryastuti, M. Rustema-Abbing, R. T. Rozenbaum, H. C. van der Mei, H. J. Busscher, S. W. Wessela, *Appl. Environ. Microbiol.* **2018**, *84*, e01035.

[5] M. J. Abraham, T. Murtola, R. Schulz, S. Páll, J. C. Smith, B. Hess, E. Lindahl, *GROMACS: SoftwareX* **2015**, *1-2*, 19.

[6] D. H. de Jong, G. Singh, W. F. D. Bennett, C. Arnarez, T. A. Wassenaar, L. V. Schäfer, X. Periole, D. P. Tieleman, S. J. J. Marrink, *Chem. Theory Comput.* **2012**, *9*, 687.

[7] T. Darden, D. York, L. Pedersen, *J. Chem. Phys*. **1998**, *98*, 10089.

[8] G. Bussi, D. Donadio, M. Parrinello, *J. Chem. Phys.* **2007**, *126*, 014101.

[9] M. Parrinello, A. Rahman, *J. Appl. Phys.* **1981**, *52*, 7182.

[10] S. Páll, B. Hess, *Comput. Phys. Commun.* **2013**, *184*, 2641.

[11] L. Schrödinger, *PyMOL the PyMOL Molecular Graphics System* **2010**.
